# Construction and characterization of recombinant human adenovirus type 5 expressing foot-and-mouth disease virus capsid proteins of Indian vaccine strain, O/IND/R2/75

**DOI:** 10.14202/vetworld.2015.147-155

**Published:** 2015-02-10

**Authors:** Ramesh Kumar, B. P. Sreenivasa, R. P. Tamilselvan

**Affiliations:** FMD Research Centre, Indian Veterinary Research Institute, Bangalore - 560 024, India

**Keywords:** foot-and-mouth disease, growth kinetics, recombinant hAd5, thermostability, virus-like particles

## Abstract

**Aim:**

Generation of recombinant human adenovirus type 5 expressing foot-and-mouth disease virus (FMDV) capsid protein genes along with full-length 2B, 3B and 3C^pro^ and its characterization.

**Materials and Methods:**

FMD viral RNA isolation, cDNA synthesis, and polymerase chain reaction were performed to synthesize expression cassettes (P1-2AB3BC^wt^ and P1-2AB3BC^m^) followed by cloning in pShuttle-CMV vector. Chemically competent BJ5183-AD-1 cells were transformed with the recombinant pShuttle-CMV to produce recombinant adenoviral plasmids. HEK-293 cells were transfected with the recombinant adenoviral plasmids to generate recombinant adenoviruses (hAd5/P1-2AB3BC^wt^ and hAd5/P1-2AB3BC^m^). Expression of the target proteins was analyzed by sandwich ELISA and indirect immunofluorescence assay. The recombinant adenoviruses were purified and concentrated by CsCl density gradient ultracentrifugation. Growth kinetics and thermostability of the recombinant adenoviruses were compared with that of non-recombinant replication-defective adenovirus (dAd5).

**Results:**

The recombinant adenoviruses containing capsid protein genes of the FMDV O/IND/R2/75 were generated and amplified in HEK-293 cells. The titer of the recombinant adenoviruses was approximately 10^8^, 10^9.5^ and 10^11^ TCID_50_/ml in supernatant media, cell lysate and CsCl purified preparation, respectively. Expression of the FMDV capsid protein was detectable in sandwich ELISA and confirmed by immunofluorescence assay. Growth kinetics of the recombinant adenoviruses did not reveal a significant difference when compared with that of dAd5. A decrement of up to 10-fold at 4°C and 21-fold at 37°C was recorded in the virus titers during 60 h incubation period and found to be statistically significant (p<0.01).

**Conclusion:**

Recombinant adenoviruses expressing capsid proteins of the FMDV O/IND/R2/75 were constructed and produced in high titers. *In vitro* expression of the target proteins in the adenovirus vector system was detected by sandwich ELISA and immunofluorescence assay.

## Introduction

Foot-and-mouth disease (FMD) is a highly ­contagious vesicular disease of cloven-hoofed animals posing a great threat to the world economy [1]. The disease is caused by FMD virus (FMDV), a highly divergent small RNA virus of the genus *Aphthovirus* under the family *Picornaviridae* [2]. There are 7 serotypes of the virus namely O, A, C, Asia-1, SAT-1, SAT-2 and SAT-3. Infection with one serotype does not produce immunity to other serotypes. Among domesticated animals, cattle, buffalo, swine, sheep and goat are susceptible to the disease. African wild buffaloes maintain SAT serotypes of the virus in oropharyngeal region and act as carriers to cloven-hoofed wildlife [3]. The disease is transmitted via contaminated air and fomites or direct contact with infected animal. After infection, the virus replicates rapidly, and viremia occurs within a day. The virus transmission occurs at 0.3-0.7 day before the appearance of clinical signs [4]. Generally, FMD is characterized by formation of vesicles on the feet, buccal mucosa and mammary glands, and drooling of saliva in the form of string. After recovery, the affected animals retain the virus in their oro-pharynx and may act as a carrier for the disease transmission to the susceptible animals.

The FMDV is 25 nm in diameter and consists of a single-stranded positive-sense RNA genome ­ sur­rounded by four structural proteins to form an icosahedral capsid [5]. FMD viral genome consists of a large single open reading frame (ORF) flanked by highly structured 5’ and 3’untranslated regions (UTR). The 5’ UTR is divided into five elements, S-fragment, poly C tract, pseudo-knots, *cis*-acting replicative element (cre), and internal ribosome entry site (IRES). The IRES serves for internal initiation of protein ­synthesis in cap-independent manner. The 3’ UTR contains a short stretch of RNA which folds into a specific stem-loop structure [6] of about 90 nucleotides, followed by a poly(A) tract of variable length. Both the UTRs play roles in virus translation and RNA replication. The ORF is translated to single polyprotein, which is subsequently processed by virus-encoded proteases (e.g. 3C^pro^) to produce four structural and eight non-structural proteins. These proteins self-assemble to form icosahedral, virus-like particles (VLPs), which contain 60 copies of each structural protein (VP1, VP2, VP3, and VP4) encapsidating the single stranded RNA genome.

The disease is endemic and prevalent in many countries in Africa, the Middle East, Asia and South America, and occurs in the form of outbreaks. In India, the disease is caused by the serotypes O, A, and Asia-1 among which the serotype O is the most prevalent (93.3%) [7]. Currently, the disease is controlled by vaccination in developing countries while developed countries follow stamping out policy [8]. Inactivated whole virus vaccine has been widely used to control and prevent the disease. However, the preparation of the inactivated virus vaccine requires costly biocontainment facilities, large quantities of live virus and cold chain maintenance, and is associated with risk of escape of the live virus from the vaccine manufacturing unit to the environment and from vaccine due to incomplete activation, and is also not suitable for differentiating infected from vaccinated animals (DIVA) strategy [1].

Various approaches have been tried to develop alternatives to the inactivated whole virus vaccine. VLPs based vaccine has received a lot of attention and considered one of the most appealing approach for contemporary vaccine design [9] due to their immunogenic properties and high safety profile for vaccine delivery platforms [10]. VLPs have been generated by use of various expression systems like vaccinia virus [11], adenovirus [12], *Escherichia coli* [13] and baculovirus [14]. VLPs of the FMDV have been produced *in vitro* by co-expression of P1-2A and 3C^pro^ [15,16]. These are structurally similar to whole virus particles but noninfectious and safe, induce efficient humoral and cellular immune response [17,18] and also suitable for DIVA strategy.

Recombinant adenoviruses have become vectors of choice for target gene delivery, expression of foreign antigens, and have been used in gene therapy, vaccination and cancer therapy [19,20]. The adenoviruses are considered as powerful vectors because of their ability to recombine in culture, high production titers, relatively high capacity for transgene insertion, efficient transduction of both quiescent and actively dividing cells, and for inducing humoral and cellular immune responses [21-23]. FMD molecular vaccine based on replication deficient human adenovirus serotype 5 (hAd5) carrying FMD capsid genes was developed and licensed for use as emergency response tool during any FMD outbreak in the USA [24]. The hAd5 carrying FMDV capsid protein antigen (P1-2A) along with 3C^pro^ have been tested in mice [17,25], guinea pigs [26], swine [17,26] and cattle [27] to protect them from homologous challenge virus. The hAd5 containing full length 2B has been reported to induce a rapid and increased FMDV-specific humoral and cellular immune responses as compared to the original vector [28,29].

Hence, considering the above facts, this study was carried out to construct and characterize hAd5 expressing capsid proteins (P1-2A) along with full-length 2B, 3B and 3C^pro^ (wild-type: 3C^wt^ and/mutant type: 3C^m^) of the FMDV O//IND/R2/75.

## Materials and Methods

### Ethical approval

All the genetic manipulation for producing recombinant adenoviruses expressing FMDV capsid proteins were carried out after obtaining necessary approval from the Institute Biosafety Committee (IBSC) and Review Committee on Genetic Manipulation (RCGM), Department of Biotechnology, Government of India.

### Cells and cell culture

The human embryonic kidney (HEK-293) cell line (# 240085, Agilent Technologies, USA) was grown in Eagle’s minimum essential medium (# 12-611Q, Lonza, Belgium) containing 10% fetal bovine serum (FBS), 25mM HEPES, non-essential amino acids (Lonza, Belgium), penicillin (100 IU/ml) and streptomycin (100 µg/ml). The cells were incubated at 37°C in a humidified CO_2_ incubator (Thermo Scientific, USA).

### Construction of the expression cassettes

Viral RNA (FMDV O/IND/R2/75) was isolated by RNeasy Mini Kit (Qiagen, Germany) and converted into cDNA using Thermoscript RT (Invitrogen, USA) as per the manufacturer’s instruction. The genes of interest (P1-2AB, 3BC^wt^ and 3BC^m^) were amplified by polymerase chain reaction (PCR) using primers enlisted in Table-1. All the PCR were performed using a reaction mix containing Phusion HF DNA polymerase (NEB, USA), Phusion buffer, dNTP, primer pair and template cDNA in a thermocycler (Applied Biosystem, USA). P1-2AB PCR was performed with the initial denaturation at 94°C/5 min, followed by 30 cycles with denaturation at 94°C/30 s, annealing at 58°C/30 s and extension at 72°C/2 min 30 s. Final extension was carried out at 72°C/10 min. 3BC^wt^ PCR was performed under the same thermal conditions except cyclic extension at 72°C/1 min and final extension at 72°C/5 min. Synthesis of 3BC^m^ was carried out in two steps. In the first step, two segments of the 3BC^m^ (3BC^1^ and 3C^2^) were synthesized using the mutagenic primers enlisted in Table-1. The amplification of above two segments was carried out with initial denaturation at 94°C/5 min, 30 cycles of denaturation at 94°C/30 s, annealing at 58°C/30 s and extension at 72°C/30 s. Final extension was carried out at 72°C for 5 min. Then, these two segments were used as a template to synthesize 3BC^m^ as per the PCR program for 3BC^wt^. All the PCR products were purified by PCR purification kit (Fermentas, USA). All the gene segments mentioned above were digested with *Spe*I enzyme (NEB, USA). Either the 3BC^wt^ or 3BC^m^ was ligated to P1-2AB by T4 DNA ligase (Thermo Scientific, USA) to construct expression cassettes P1-2AB3BC^wt^ and P1-2AB3BC^m^. Both the expression cassettes were similar except glycine (G) at position 38 and phenylalanine (F) at position 48 of 3C^wt^ were replaced with serine (S) in 3C^m^.

**Table-1:** PCR primers for synthesis of expression cassettes P1-P12AB3BC^wt^ and P1-2AB3BC^m^ of FMDV O/IND/R2/75.

Primer	Functional region	Sequence (5’→3’)
SalI-VP4-F	P1-2ABC^1^	TGGTCGACATGGGAGCYGGGCAATCCAG
SpeI-2C-R	P1-2ABC^1^	TGACTAGTTATGTCRTTRATGTCACGTGCTTTGAG
SpeI-3A-F	3A^1^BC	TGACTAGTGAGCCCACCAAACCCGTG
HindIII-3C639-R	3A^1^BC	TGAAGCTTACTCGTGGTGTGGTTCGG
G38F48S-F2^2^	3C	AGCACTGCCTACCTCGTGCCTCGTCATCTTTCCGCAGAGAAG
G38S-F48S-R2^2^	3C	GGAAAGATGACGAGGCACGAGGTAGGCAGTGCTGAACACTCC

PCR=Polymerase chain reaction, FMDV=Footandmouth disease virus,

1 represents partial sequence containing few nucleotides of the respective gene (e.g., 3A and 2C),

2 represents the primers used for PCR mutagenesis

### Cloning of the gene of interest (GOI)

The expression cassettes were cloned into pShuttle-CMV vector (# 240007, Agilent Technologies, USA) between *Sal*I and *Hin*dIII sites flanked by CMV promoter and SV40 poly(A) signal. Top 10 *E. coli* cells (Life technologies, USA) were transformed with the recombinant pShuttle vectors (pSH-CMV/P1-2AB3BC^wt^ and pSH-CMV/P1-2AB3BC^m^). The transformed Top 10 cells were spread-plated on Luria Burtony agar (LB powder, Affymetrix, USA) containing kanamycin (50 µg/ml) and incubated at 37°C for 18-20 h. The Top 10 cells containing the recombinant pShuttle vectors are kanamycin resistant and grow on LB agar containing the antibiotic while non-recombinant Top 10 cells die out. The potential recombinant colonies were tested for GOI by colony PCR. One of the positive colonies was inoculated in LB broth containing kanamycin and incubated at 37°C in a shaker incubator (Thermo Scientific, USA) to amplify the plasmid. The plasmids were isolated by Plasmid Miniprep kit (# K0502, Fermentas, USA) as per the manufacturer’s instruction. The integrity of GOI was checked by PCR and RE digestion, and confirmed by sequencing. The sequencing of the target genes was carried out in an automated DNA Sequencer (Perkin Elmer, USA).

### Construction of recombinant adenovirus plasmids

Electroporation competent BJ5183-AD-1 cells (#200157, Agilent Technologies) were used to prepare chemically competent cells as per the protocol described by Sambrook and Russel [30]. The recombinant pShuttle-CMV vectors were linearized with *Pme*I enzyme (NEB, USA) and used to transform the chemically competent BJ5183-AD-1 cells to generate recombinant adenovirus plasmids (pAd/P1-2AB3BC^wt^ and pAd/P1-2AB3BC^m^) by homologous recombination. The transformed BJ5183-AD-1 cells were grown on LB agar containing kanamycin (50 µg/ml) and incubated at 37°C for 18 h. The resultant colonies were tested for GOI in colony PCR and the positive colonies were grown in LB broth overnight at 37°C in a shaker incubator at 220 rpm followed by plasmid isolation. The plasmids were further amplified in the Top10 cells growing in Teriffic broth (Invitrogen, USA) containing kanamycin and isolated by Plasmid Midi Kit (Qiagen, Germany) as per the protocol described by the manufacturer.

### Generation of recombinant adenovirus vectors

The adenovirus plasmids were digested with *Pac*I enzyme (Fermentas, USA) before transfection to expose the inverted terminal repeats. The HEK-293 cells were cultured in 6 well tissue culture plates (Nunc, Denmark) for 24 h to achieve approximately 50% confluency. Transfection of HEK-293 cells with the above adenovirus plasmids (4 µg of the plasmid DNA per well) was performed by using Virapack Transfection Kit (#200488, Agilent Technologies, USA). The transfected cells were observed daily up to 10 days for the appearance of virus induced plaques. Plaques, which appeared early, were individually picked up carefully and pelleted by centrifuging at 200 g for 10 min. The cells were re-suspended in Dulbecco’s phosphate buffer saline (DPBS) containing Ca^++^ and Mg^++^ and freeze-thawed thrice to release the virus. Then the virus-cell suspension was centrifuged at 5000 g for 10 min and supernatant containing the virus was collected and stored at −80°C. The plaque derived viruses were titrated, and the viruses having the higher titer were selected to prepare seed viruses. The seed viruses were used for further amplification of the virus. The viruses were serially passaged by infecting the HEK-293 cells at a multiplicity of infection (MOI) of 0.1.

### Immunohistochemistry and sandwich-ELISA

An indirect immuno-fluorescence assay was performed to detect expression of the target gene. The HEK-293 cells were grown in 24 well tissue culture plate as monolayer and infected with the recombinant viruses (passage 2) at an MOI of 0.1. When cytopathic effect (CPE) appeared as plaques, the infected cell monolayer was used for the immunofluorescence assay. The medium was discarded, and the monolayer was washed twice with PBS and air dried at room temperature. The cells were fixed with chilled acetone-methanol (1:1). Blocking was done by incubating the cells with 3% bovine serum albumin in PBS at room temperature for 30 min. Rabbit anti-FMDV serotype O serum (1:200 diluted in PBS) was used to probe expressed FMDV antigen followed by washing with PBS containing 0.05% Tween-20 (PBST). The cells were then stained with Alexa Fluor 488-conjugated goat anti-rabbit IgG (1:2000 diluted in PBS). The unbound antibodies were removed by washing with PBST and the cells were observed under a fluorescence microscope (Nikon, Japan).

An antigen capture ELISA (indirect sandwich ELISA) was also used to detect FMDV antigens as per the protocol described in OIE manual [3]. Test samples were diluted 1:7 in DPBS before testing for the target antigen. Rabbit antiserum to 146S antigen of FMDV O/IND/R2/75 was used for capturing expressed antigens. The plates were read at 492 nm in ELISA reader (Tecan, USA). An absorbance reading > 0.1 above the background indicates a positive reaction [3] while < 0.1 is considered as negative.

### Titration of the adenovirus

Serial 10-fold dilutions of the virus were prepared in the culture media. 100 µl of each dilution was added to 10 wells of a 96 well tissue culture plate, followed by addition of the HEK-293 cells prepared in the media containing 6% FBS. The plate was incubated at 37°C in CO_2_ incubator for 10 days and observed daily for CPE. Virus titer was calculated as per the method described by Reed and Muench [31].

### Purification of the recombinant adenoviruses

The recombinant adenoviruses were purified by CsCl density gradient ultracentrifugation in two steps. First, the clarified cell lysate containing the adenovirus was layered on CsCl discontinuous gradient and centrifuged at 23000 rpm at 15°C for 2 h in an ultracentrifuge (Sorvall WX Ultra Series Centrifuge, Thermo Scientific, USA). In second step, the adenovirus obtained after discontinuous gradient purification was subjected to continuous gradient centrifugation at 23000 rpm at 15°C for 20 h for further purification and concentration. The viruses were collected, dialyzed and stored at −80°C.

### Growth kinetic study

The HEK-293 cell monolayer grown in 25 cm^2^ tissue culture flasks (Nunc) was infected separately with the recombinant adenoviruses or non-recombinant replication-defective human adenovirus 5 (dAd5, available at IVRI, Bangalore) at an MOI of 0.1. The viruses were allowed to adsorb for 1 h and then the monolayer was washed twice with PBS. The infected cells were harvested at 0 h (immediately after adsorption) and at every 12 h intervals post infection up to 5 days and frozen at −80°C. Titer of each sample was determined in duplicate and mean value was used to study the growth kinetics of the adenoviruses.

### Thermostability determination

The CsCl ultra-purified recombinant viruses and dAd5 were incubated in microcentrifuge tubes at 4°C and 37°C for 60 h. The samples were collected at 0 h and at every 12 h interval and frozen at −80°C until used for virus titration. Titer of each sample was calculated in duplicate, and the mean of the two values was used for comparison.

### Statistical analysis

ELISA absorbance readings from duplicate wells were used to calculate mean +/- SD upon testing of expressed antigens collected at various time points. Regression analysis was performed to correlate incubation temperature to the incubation time using SAS 9.3.

## Results

### Analysis of the target genes

**Figure-1:**
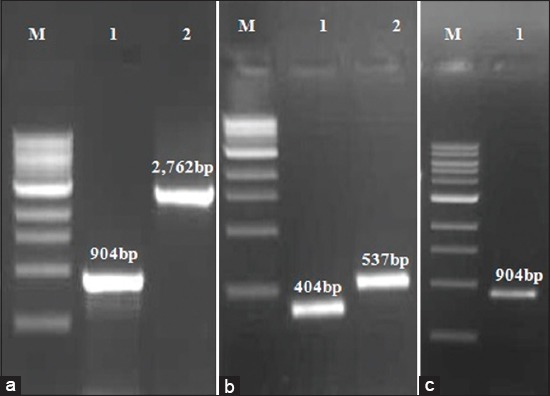
Polymerase chain reaction (PCR) amplification of capsid protein genes of foot-and-mouth disease virus O/IND/R2/75. Lane M: 1 kb DNA ladder (# N3232S, NEB, USA); (a) lane 1: 3BC^wt^; lane 2: P1-2AB. (b) Mutagenic PCR products, lane 1: 3BC^1^; lane 2: 3C^2^. (c) Overlap PCR product, lane 1: 3BC^m^.

**Figure-2:**
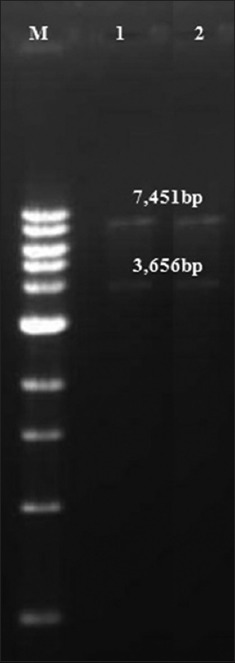
Restriction digestion of recombinant pSH-CMV vector with *Sal*I-HF and *Hin*dIII. Lane M: 1kb DNA ladder (# N3232S, NEB, USA); lane1: pSH-CMV/P1-2AB3BC^wt^; lane2: pSH-CMV/P1-2AB3BC^m^. The smaller segment represents the cloning of the gene of interest.

Products of desired size 2,762 bp (P1-2AB) and 904 bp (3BC^wt^) were detected in agarose gel electrophoresis of the PCR products (Figure-1a). In mutagenic PCR, two fragments of size 404 bp and 537 bp were amplified (Figure-1b) and subsequently used as a template to synthesize and amplify 3BC^m^ (904 bp) by overlap PCR (Figure-1c). In colony PCR, out of 5 colonies screened, 3 each were found positive for P1-2AB3BC^wt^ and P1-2AB3BC^m^. Among the 3 positive colonies, one was selected for amplification of the recombinant pSH-CMV. The expression cassettes (3,656 bp) were found intact in recombinant pSH-CMV vectors (pSH-CMV/P1-2AB3BC^wt^ and pSH-CMV/P1-2AB3BC^m^) by restriction enzyme analysis (Figure-2). Sequencing results (not provided) confirmed that both the expression cassettes were similar with two desired induced mutations in mutant type when compared with wild type parent genome.

### Analysis of the recombinant adenoviral plasmids

**Figure-3:**
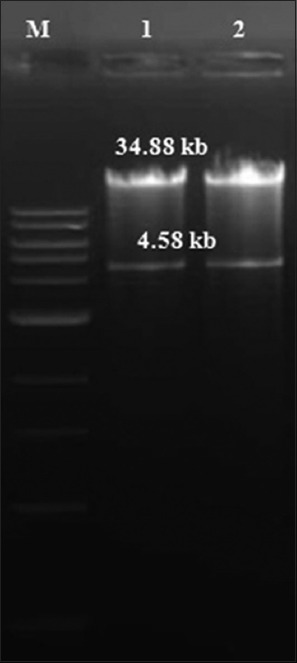
*Pac*I digestion pattern of recombinant pAd5 plasmids. Lane M: 1 kb DNA ladder (# N3232S, NEB, USA); lane1: pAd5/P1-2AB3BC^wt^; lane2: pAd5/P1-2AB3BC^m^. Smaller fragment of size 4,588 bp indicates that homologous recombination in BJ5183-AD-1 cells took place at ori site. Larger fragment is used to transfect HEK-293 cells to generate recombinant hAd5.

Growth of the transformed BJ-5183 cells on LB agar resulted into three types of colonies *viz*., small, intermediate and large. Small colonies, being the potential recombinants, were tested for GOI by colony PCR and found to be positive. *Pac*I digestion of the recombinant adenovirus plasmids resulted in two fragments of size about 34.9 kb and 4.5 kb (Figure-3). The larger fragment represents linearized adenovirus plasmid while smaller one indicates that the recombination in the transformed BJ5183-AD-1 cells took place in the right arms and origin of replications (AdEasy™ XL adenoviral vector system).

### Recombinant viruses expressing target antigen

**Figure-4:**
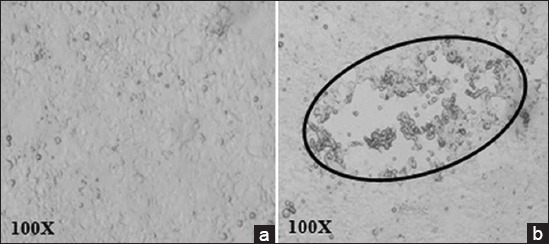
Transfection of HEK-293 cells with recombinant pAdEasy1. (a) Uninfected HEK-293 cells at confluency. (b) HEK-293 cells 5 day post transfection, CPE was observed as rounding, detachment and coalescing of the cells (plaque). The encircled cells were harvested and used to prepare seed virus.

**Figure-5:**
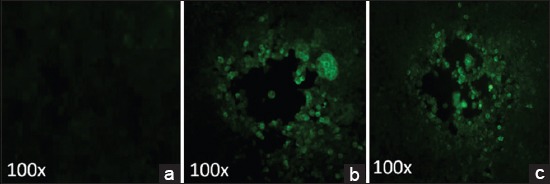
Fluorescence microscopy of HEK-293 cells infected with recombinant hAd5 expressing FMDV capsid proteins. (a) Uninfected cells without fluorescence (b) Cells infected with hAd5/P1-2AB3BC^wt^ and (c) hAd5/P1-2AB3BC^m^ showing fluorescence which indicates the expression of FMDV type O capsid proteins.

In comparison to uninfected HEK-293 cells which did not show any visible changes (Figure-4a), CPE was observed in the form of plaques as early as 4 day post transfection of HEK-293 cells with pAd5/P1-2AB3BC^m^ vector (Figure-4b), which indicates generation of the recombinant adenovirus. The adenovirus hAd5/P1-2AB3BC^wt^ was generated 5-day post transfection. The expression of FMD capsid proteins was detected in indirect immunofluorescence assay (Figure-5) and FMDV type-specific sandwich ELISA (Table-2). All the plaque samples were found to be positive for FMDV type O antigen in the ELISA.

**Table-2:** Virus titration and expression analysis of plaque derived viruses.

hAd5/P1-2AB3BC^wt^			hAd5/P1-2AB3BC^m^		
	
Sample ID	Virus titer (log_10_TCID_50_/ml)	Mean O.D±SD (492 nm)	Sample ID	Virus titer (log_10_TCID_50_/ml)	Mean OD±SD (492 nm)
Plaque 1	6.69	0.124±0.001	Plaque 1	7.50	0.239±0.001
Plaque 2	6.23	0.425±0.003	Plaque 2*	8.33	0.273±0.004
Plaque 3	5.00	0.161±0.006	Plaque 3	8.00	0.291±0.005
Plaque 4*	7.50	0.248±0.002	Plaque 4	5.67	0.496±0.006
Plaque 5	6.50	0.183±0.003	Plaque 5	7.50	0.144±0.004
Plaque 6	5.67	0.361±0.006	Plaque 6	6.69	0.378±0.002
Plaque 7	6.23	0.215±0.007	Plaque 7	8.00	0.261±0.002
Plaque 8	7.38	0.305±0.003	Plaque 8	7.56	0.201±0.006
Plaque 9	7.32	0.325±0.003	Plaque 9	6.23	0.210±0.001
Plaque 10	6.00	0.219±0.002	Plaque 10	8.00	0.239±0.006

* Denotes that this virus sample was selected for preparation of seed virus stock

### Quantification of the viruses at different passage level

The titer of the primary stock viruses is given in Table-2. Plaque 4 of the hAd5/P1-2AB3BC^wt^ virus and plaque 2 of the hAd5/P1-2AB3BC^m^ were found to contain highest virus titer among all plaques and selected for further amplification and characterization of the viruses. The virus titers of approximately 10^8^, 10^9.5^ and 10^11^ TCID_50_/ml were recorded in supernatant, cell lysate, and CsCl purified and concentrated preparation of the recombinant viruses, respectively. Titers of the selected viruses up to passage 6 are given in Table-3.

**Table-3:** Titration of the recombinant hAd5 viruses at various passage level.

Sample ID	Virus titer (log_10_TCID_50_/ml)					

	P1	P2	P3	P4	P5	P6
hAd5/P1-2AB-3BC^wt^						
Cell lysate	8.67	9.60	10.00	9.50	9.8	9.67
CsCl purified	NA	10.70	10.77	10.67	10.7	10.60
hAd5/P1-2AB3BC^m^						
Cell lysate	9.00	9.85	9.60	9.77	9.50	9.65
CsCl purified	NA	10.79	10.33	10.50	10.69	10.42

NA=Not applicable, means that the virus was not purified

### Virus growth kinetics and thermal stability

**Figure-6:**
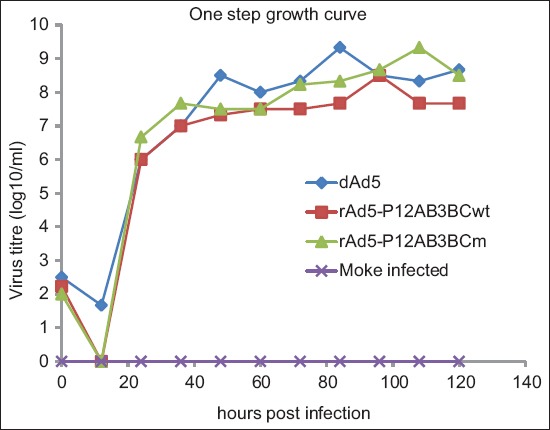
Growth kinetics of the hAd5/P1-2AB3BC^wt^, hAd5/P1-2AB3BC^m^ and dAd5. HEK-293 cells were infected with the viruses at a multiplicity of infection of 0.1 and samples were harvested at 12 h inverval. Titer of the samples was calculated as per Reed and Muench (1938) and the graph was plotted.

Titers of the adenovirus samples, obtained in the growth kinetics study, are given in Table-4 and used to draw virus growth kinetic curve as shown in Figure-6. At 12 h post infection (hpi), no virus was detected in both hAd5/P1-2AB3C^wt^ and hAd5/P1-2AB3C^m^ infected samples while virus replication was evident in dAd5. An increase in titer of all the three adenoviruses could be observed during first 24 to 48 hpi. Titer of the viruses remained more or less constant during 48-120 hpi with slight variation. The CPE was completed in 4-5 days post infection while mock-infected HEK-293 cells remained intact.

**Table-4:** Virus titration result of the adenovirus samples for growth kinetic study.

Sample ID	Virus titer (log_10_TCID_50_/ml)			

	dAd5	hAd5/P1-2AB3C^wt^	hAd5/P1-2AB3C^m^	Mock infected
0 hpi	2.50	2.23	2.00	0.00
12 hpi	1.67	0.00	0.00	0.00
24 hpi	6.00	6.00	6.67	0.00
36 hpi	7.00	7.00	7.67	0.00
48 hpi	8.50	7.33	7.50	0.00
60 hpi	8.00	7.50	7.50	0.00
72 hpi	8.33	7.50	8.23	0.00
84 hpi	9.33	7.67	8.33	0.00
96 hpi	8.50	8.50	8.67	0.00
108 hpi	8.33	7.67	9.33	0.00
120 hpi	8.67	7.67	8.50	0.00

**Figure-7:**
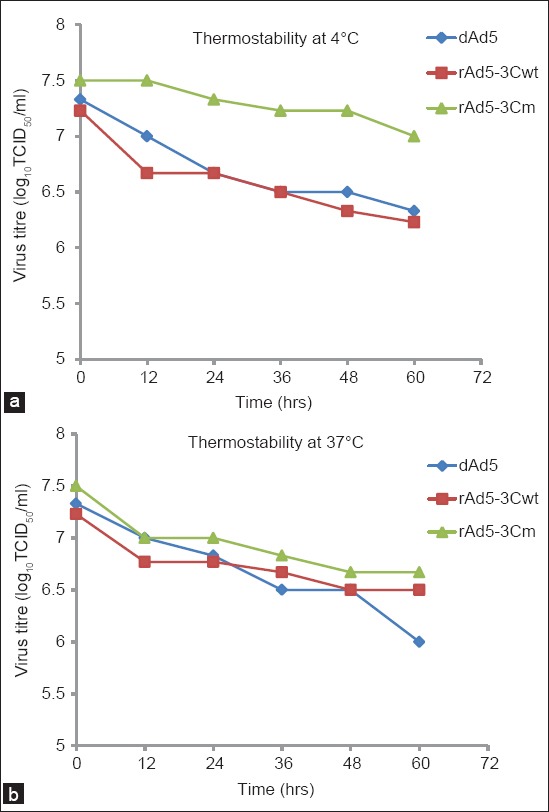
Thermostability assay of the hAd5/P1-2AB3BC^wt^, hAd5/P1-2AB3BC^m^ and dAd5. The adenoviruses were incubated at 4°C and 37°C and samples were collected at 12 h interval up to 60 h. Titer of the samples was calculated in duplicate and mean of the titers was used to correlate the thermal stability of the adenoviruses (virus titer at Y-axis) to the incubation time (X-axis). (a) Thermal stability of the viruses at 4°C, and (b) at 37°C is shown.

A slight decrease in the virus titer was observed during the thermal stability test. Titer of the recombinant viruses decreased up to 21-fold at 37°C and up to 10-fold at 4°C during 60 h of incubation as presented in Table-5 and demonstrated in Figure-7. A linear regression analysis showed that the virus titer was negatively correlated to the incubation time (at 37°C and 4°C) and found to be statistically significant (p<0.01).

**Table-5:** Relationship between virus titer and duration of exposure at 4°C and 37°C.

Time^a^	Virus titer (log_10_TCID_50_/ml)											

	dAd5 virus				hAd/P1-2AB3BC^wt^				hAd/P1-2AB3BC^m^			
		
	37°C		4°C		37°C		4°C		37°C		4°C	
					
	Titer	R^b^	Titer	R^b^	Titer	R^b^	Titer	R^b^	Titer	R^b^	Titer	R^b^
0	7.33	1	7.33	1	7.23	1	7.23	1	7.50	1	7.50	1
12	7.00	2.13	7.00	2.13	6.77	2.88	6.67	3.63	7.00	3.16	7.50	1
24	6.83	3.16	6.67	4.57	6.77	2.88	6.67	3.63	7.00	3.16	7.33	1.47
36	6.50	6.76	6.50	6.76	6.67	3.63	6.50	5.37	6.83	4.67	7.23	1.86
48	6.50	6.76	6.50	6.76	6.50	5.37	6.33	7.94	6.67	6.76	7.23	1.86
60	6.00	21.38	6.33	10	6.50	5.37	6.23	10	6.67	6.76	7.00	3.16

a Represents duration of exposure (in hours) of the sample,

b represents fold decrease in the virus titer with respect to the titer at 0 h

## Discussion

FMD is an important disease of transboundary importance affecting cloven-hoofed animals worldwide [1]. Seven serotypes of FMDV with many subtypes in each serotype and no cross-protection against each other make the disease control difficult. Serotype O virus is antigenically poor compared to A and Asia-1 serotypes, and thus large amount of serotype O antigen is required to achieve same level of protection in the inactivated vaccine [32]. Vaccines based on inactivated whole viruses emulsified with adjuvant have been effectively used to control and prevent the disease in enzootic countries. But due to several limitations of the inactivated vaccine, various alternate approaches have been tried to develop a better vaccine candidate [33].

The replication-defective human adenovirus type 5 has been proved as a potential gene delivery vehicle [29,34,35]. P1-2A-3C being a basic expression cassette of FMD virion is responsible for empty capsid particle formation [15] while 2B has been reported to induce rapid immune responses [28,29]. In the present study, incorporation of 3B downstream of 2B in the expression cassette provides two additional cleavage sites for 3C^pro^ besides increasing the genome size of the vector that may reduce the toxicity of 3C^pro^ to the infected cells. It has been demonstrated that the length of the inserted gene affects the genetic stability of the vector and a genome size <75% or > 105% of the wild-type (wt) genome tends to rearrange to achieve a size closer to wt genome [36,37] and may recombine to the packaging cell line genome to generate replication competent adenovirus. In our study, the genome size of recombinant hAd5 (34.8 kb) was about 91% of the wt genome which is optimum for genome packaging.

It is widely believed that wt 3C^pro^ is toxic to the cells and adversely affects the expression of the recombinant proteins [38]. The present study reveals that the mutations in the wt 3C^pro^ have not significantly affected the virus titer and expression of target antigen. This may suggest that mutations in 3C^pro^ did not provide any replicative advantage to the virus. Our result demonstrated that the expression level of the target protein was not significantly correlated to the virus titer (Pearson correlation coefficient r=0.15441, p=0.6702 for hAd5/P1-2AB3BC^wt^ and r=-0.5520, p=0.0980 for hAd5/P1-2AB3BC^m^) as depicted in Table-2. Further study, involving electron microscopy and animal immunization are necessary to confirm the formation of empty capsid particles and immunogenic potential of the recombinant adenovirus vaccines.

The growth kinetics of both the recombinant viruses was more or less similar and slightly different from that of dAd5. No virus particle was seen at 12 hpi in the HEK-293 cells infected with the recombinant adenoviruses indicating that the whole virus inoculum was adsorbed and the virus particles were in eclipse phase while the presence of virus activity was evident in the cells infected with dAd5. Generation time for the recombinant adenoviruses was recorded as >12 h while for dAd5 it was ≥12 h. The delayed appearance of progeny viruses in the cells infected with recombinant hAd5 viruses may have been due to increased size of the genome compared to dAd5. The growth kinetics of the viruses was found to be similar to those of the previous study involving hAd5 [39] and hAd3 [40]. Maximum growth was recorded during first 48 h which decreased later on which may be due to unavailability of sufficient healthy cells for a further round of virus replication. Our result of thermal stability of the viruses is in agreement with those of previous studies [41,42] and found to be statistically significant. The dAd5 was less stable at 37°C compared to the recombinant viruses while hAd5/P1-2AB3BC^m^ was more stableat 4°C as compared to hAd5/P1-2AB3BC^wt^. In general, the virus titer decreased up to 10-fold at 4°C and 21-fold at 37°C after 60 h of incubation. In a study, Xue *et al*. [40] demonstrated that thermostability of the hAd5 at 45°C decrease more rapidly.

## Conclusions

The recombinant adenoviruses carrying capsid protein-coding gene of FMDV O/IND/R2/75 were generated and amplified to a high titer. The target protein was efficiently expressed in the adenovirus vector expression system as tested by the immunofluorescence assay and ELISA. The recombinant adenoviruses exhibited stability at 37°C for 60 h with a slight decrease in the titer. Further studies on *in vitro* detection of recombinant adenovirus expressed FMDV empty capsid particles by electron microscopy and animal immunization are necessary to evaluate immunological potential of the adenovirus vectored FMD vaccines.

## Author’s contributions

BPS and RK planned and designed the research work. All the experiments were performed by RK under the supervision of BPS. Sandwich ELISA and statistical analysis were carried out by RPTS. The manuscript was prepared by RK and reviewed by BPS and RPTS. All the authors approved the final manuscript.
